# Silencing of Activity During Hypoxia Improves Functional Outcomes in Motor Neuron Networks *in vitro*

**DOI:** 10.3389/fnint.2021.792863

**Published:** 2021-12-16

**Authors:** Vegard Fiskum, Axel Sandvig, Ioanna Sandvig

**Affiliations:** ^1^Department of Neuromedicine and Movement Science, Faculty of Medicine and Health Sciences, Norwegian University of Science and Technology, Trondheim, Norway; ^2^Department of Neurology, St. Olav’s Hospital, Trondheim University Hospital, Trondheim, Norway; ^3^Department of Pharmacology and Clinical Neurosciences, Division of Neuro, Head, and Neck, Umeå University Hospital, Umeå, Sweden; ^4^Department of Community Medicine and Rehabilitation, Umeå University, Umeå, Sweden

**Keywords:** hypoxia, multielectrode array (MEA) recording, motor neuron disease, activity-dependent mechanisms, longitudinal, network activity

## Abstract

The effects of hypoxia, or reduced oxygen supply, to brain tissue can be disastrous, leading to extensive loss of function. Deoxygenated tissue becomes unable to maintain healthy metabolism, which leads to increased production of reactive oxygen species (ROS) and loss of calcium homoeostasis, with damaging downstream effects. Neurons are a highly energy demanding cell type, and as such they are highly sensitive to reductions in oxygenation and some types of neurons such as motor neurons are even more susceptible to hypoxic damage. In addition to the immediate deleterious effects hypoxia can have on neurons, there can be delayed effects which lead to increased risk of developing neurodegenerative diseases such as amyotrophic lateral sclerosis (ALS), even if no immediate consequences are apparent. Furthermore, impairment of the function of various hypoxia-responsive factors has been shown to increase the risk of developing several neurodegenerative disorders. Longitudinal assessment of electrophysiological network activity is underutilised in assessing the effects of hypoxia on neurons and how their activity and communication change over time following a hypoxic challenge. This study utilised multielectrode arrays and motor neuron networks to study the response to hypoxia and the subsequent development of the neuronal activity over time, as well as the effect of silencing network activity during the hypoxic challenge. We found that motor neuron networks exposed to hypoxic challenge exhibited a delayed fluctuation in multiple network activity parameters compared to normoxic networks. Silencing of activity during the hypoxic challenge leads to maintained bursting activity, suggesting that functional outcomes are better maintained in these networks and that there are activity-dependent mechanisms involved in the network damage following hypoxia.

## Introduction

Hypoxia is a condition of reduced oxygen supply to brain tissue that can occur in a variety of circumstances, from low atmospheric oxygen to reduced blood flow. It is distinguished from anoxia, a more adverse condition of no oxygen supply, which is not considered in this study. Low oxygen levels can have severe adverse effects on affected tissue, including enhanced production of reactive oxygen species (ROS), impaired ATP production and loss of calcium homoeostasis (Kristian and Siesjo, 1998), all of which can lead to tissue damage and cell death. Neuronal tissue has a very high energy consumption, which makes it particularly vulnerable to hypoxic insult (Hossmann, 1999), for example following a stroke or respiratory arrest. This vulnerability is not limited to the immediate and drastic brain damage often associated with sustained reduction of oxygen supply to the brain. It can manifest in more subtle ways, notably increased risk of developing neurodegenerative disorders (Sun et al., 2006; Guglielmotto et al., 2009; Bhatia et al., 2017) as well as aggravate pathological processes in patients with existing neurodegenerative disease and animal models of neurodegenerative disease (Zhang et al., 2019). Compounding the link between hypoxia and neurodegenerative disorders is also a growing body of evidence that genetic traits which impair proper function of various hypoxia-responsive elements can increase the risk of developing neurodegenerative disorders, including Parkinson’s disease (PD) and Amyotrophic lateral sclerosis (ALS) (Greenway et al., 2006; van Es et al., 2011; Zou et al., 2012).

Hypoxia causes neurotoxic effects which may be the consequence of different mechanisms, both activity-dependent and activity-independent. Changes in neuronal excitability are widely reported in both dissociated neuronal cell cultures and brain slice preparations, albeit with contradictory findings. For example, some studies have found that hypoxia leads to neuronal hyperexcitability in Schaffer collaterals of rat hippocampal slices (Schiff and Somjen, 1985) and CA1 neurons in rat hippocampal slices (Godukhin et al., 2002), while other studies have reported decreased excitability in CA1 neurons in mouse hippocampal slices (Gu and Haddad, 2001) and dissociated mouse hippocampal neurons (Gavello et al., 2012). There may also be cell type-specific vulnerabilities which contribute to this deviance in findings, such as the reported vulnerability of inhibitory neurons (Turovsky et al., 2013). The influx of calcium into hypoxic neurons has been widely implicated in the neurotoxic process (Kristian and Siesjo, 1998), and NMDA-receptors appear to be an important avenue of Ca^2+^ influx due to their proximity to mitochondria, which act as intracellular calcium stores (Starkov et al., 2004). Blocking NMDA-receptors has been shown to reduce the formation of ROS (Lafon-Cazal et al., 1993). Activation of GABA-A receptors has also been shown to ameliorate oxidative damage during hypoxia due to Cl^–^ influx in rodents (Zhao et al., 2003; Kumar and Goyal, 2008), and inhibition of activity of hippocampal neurons with leptin also leads to improved outcomes following hypoxia (Gavello et al., 2012). Together these results suggest an activity-dependent mechanism for neuronal damage following hypoxia.

Although neuronal tissue is susceptible to hypoxia in general, there are notable differences in how different neuronal populations respond to hypoxia, and some populations are clearly more vulnerable than others. However, most of the research on neuronal susceptibility examines the vulnerable hippocampal neurons, while other vulnerable groups such as motor neurons are less frequently studied. However, not only are motor neurons particularly susceptible to hypoxia (Watzlawik et al., 2015), but repeated exposure to hypoxic conditions also appears to greatly increase the risk of developing motor neuron disorders like ALS (Vanacore et al., 2010). Motor neurons are highly energy demanding, and findings indicate that motor neurons with ALS pathology exhibit reduced mitochondrial respiration and oxidative ATP production, with a shift toward increased glycolysis (Hor et al., 2021) which can contribute to overall energy deficits which contribute to disease progression (Dupuis et al., 2011). This mirrors the effects of hypoxia, where mitochondrial activation of hypoxia inducible factor 1α (HIF-1α) in response to reduced concentration of oxygen leads to inhibition of oxidative phosphorylation and increased production of ATP through glycolysis, resulting in an overall energy deficit compared to normoxic conditions (Solaini et al., 2010). This makes the study of the responses of motor neurons to hypoxic challenge also relevant to studies of ALS, both through shared pathways and as a potential environmental contributor to the disease.

Studies of neuronal activity are underutilised in the study of hypoxia exposure, especially with regard to the effects on broader network dynamics. This study uses *in vitro* motor neuron networks on multielectrode arrays (MEAs) to examine responses to hypoxic insult in a longitudinal perspective. It considers the effects of network activity during the episode in maintaining healthy network function following hypoxia.

## Materials and Methods

### *In vitro* Neural Networks

**FIGURE 1:**
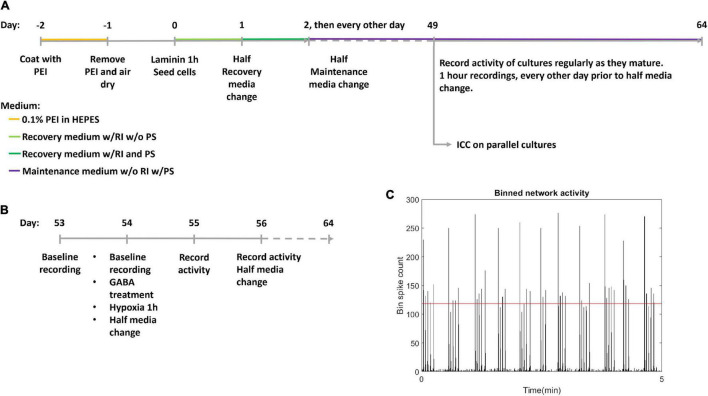
Experiment timeline. **(A)** The experimental timeline. Cells were seeded on day 0 after coating with polyethyleminine (PEI) and laminin. RI: Rock inhibitor Y-27623 2HCL, PS, Penicillin-Streptomycin antibiotics; ICC, Immunocytochemistry. **(B)** Detailed timeline of hypoxic challenge. Following day 56 activity was recorded every day, and media was half replaced every other day. **(C)** Network bursts were identified by bins of high firing rate, with the threshold indicated by the red horizontal line.

Human motor neuron progenitors derived from a healthy donor (ax0078) were purchased from Axol Bioscience. The cells were seeded on two Axion Biosystems 48-well Cytoview MEA plates (M768-tMEA-48B) and two 8-well chambered slides (Ibidi 80841). Prior to seeding, all culture wells were coated with 0.05% polyethylenimine (PEI) diluted in HEPES (both Sigma-Aldrich). MEA wells were each coated with 70 μL while slide wells were coated with 250 μL, before being left overnight in an incubator at 37°C and 5% CO_2_. Water reservoirs in the MEA plates were filled with distilled water to reduce evaporation. The next day, the PEI was removed, and the wells were rinsed four times with distilled water, before they were left to air dry at room temperature overnight. The wells were then coated with natural mouse laminin (Thermo Fisher Scientific), at 20 μg/mL, diluted in PBS. MEA wells were each coated with 70 μL while slide wells were coated with 250 μL. Cells were plated immediately after removing the laminin, without washing. Each MEA well was seeded with 25,000 motor neuron progenitors and 2,500 human astrocytes (Thermo Fisher Scientific Gibco K1884, lot 1948466) in mixed suspension, using Axol Motor Neuron Recovery media (ax0071) supplemented with retinoic acid at 0.1 μM and Y-27623 2HCL at 10 μM according to the supplier’s instructions. Each slide well was seeded with 50,000 motor neuron progenitors and 5,000 human astrocytes in the same media. The ratio of neurons to astrocytes was based on recommendations from Axion Biosystems to ensure trophic support for the neuron progenitors without interfering with electrophysiological recordings. The MEAs and slides were left in the incubator for 1 h before adding more media to reach a final volume of 300 μL in each MEA well and 400 μL in each slide well. The next day, half of the cell media volume was replaced with Axol Motor Neuron Recovery media supplemented with retinoic acid, Y-27623 2HCL and 1% Pen-Strep. The day after, and every other day for the duration of the experiment, half of the media volume was replaced with Axol Motor Neuron Maintenance media (ax0072) supplemented with 10 ng/mL CNTF, 5 ng/mL BDNF, 0.5 μM retinoic acid and 1% Pen Strep. A timeline for coating, seeding and media composition is shown in Figure 1A.

### Electrophysiological Recordings

Recordings of electrical activity were made using an Axion Maestro acquisition tool, with temperature of 37°C and 5% CO_2_, recording 1 h of baseline activity at a sampling rate of 12.5 kHz. Each MEA well recorded from 16 electrodes. After being placed in the Axion Maestro, neural networks were left for 1 h to allow activity to stabilise before recordings were started. The first recording was made at 49 DIV and subsequent recordings were made every other day until the neural networks reached 53 DIV, and daily after this. Data analysis was based on Axion spike detection using a dynamic threshold detection of 7 standard deviations. Spike times were exported *via* Neuroexplorer 5 to Matlab R2019b and analysed using custom scripts, see section “Data Analysis”. Due to a technical fault in the acquisition process, the recording of activity at 58 DIV was not stored for the hypoxic plate (data point omitted), and the recording of the non-hypoxic plate at 58 DIV only stored 23 min of the 1 h planned recording.

### Immunocytochemistry and Imaging

To confirm motor neuron identity as well as expression of relevant neurotransmitters, parallel neural networks derived from the same progenitors were tested by immunocytochemistry (ICC) at 49 DIV for the markers in Table 1, as well as nuclear staining with Hoechst (bisBenzimide H 33342 trihydrochloride, 14533, Sigma-Aldrich, 1:10,000). Prior to staining, cells were fixed in 4% Paraformaldehyde in PBS, before blocking was carried out with 5% Goat serum and 0.6% Triton-X in PBS. Primary and secondary antibody staining was done using 2.5% Goat serum and 0.3% Triton-X in PBS at the above indicated antibody dilutions.

**TABLE 1:** Immunocytochemistry (ICC): Markers, antibodies and concentrations.

Marker	Antibody catalogue number	Concentration
Islet1	Ab109517	1:250
NeuN	Ab104224	1:1,000
MAP2	Ab5392	1:2,000
HB9	Ab221884	1:25
ChAT	Ab34419	1:100
GABA-b receptor 1	Ab55051	1:100
Glutamate receptors type 2 and 3	Ab27225	1:500
Heavy neurofilament	Ab4680	1:1,000

*All antibodies were purchased from Abcam.*

Images were acquired with an EVOS M5000 microscope (Invitrogen Thermo Fisher Scientific) with DAPI (AMEP4650), CY5 (AMEP4656), GFP (AMEP4651), and TxRed (AMEP4655) LED light cubes and an Olympus UPLSAP020x lens, 20x/0.75 NA (N1480500). All image processing was done in Fiji/ImageJ.

### Hypoxic Challenge

Two multiwell MEA plates; hypoxic condition and normoxic control, plated with 24 and 21 motor neuron networks of healthy motor neurons, respectively, aged 54 DIV were used. Within the hypoxic group and the normoxic control, half the motor neuron networks were inhibited with GABA (12 networks in the hypoxic group and 11 in the normoxic control group) while the remaining networks were uninhibited (12 networks in the hypoxic group and 10 in the normoxic control group), prior to exposure to hypoxic challenge at 1% O_2_ and 5% CO_2_ for 1 h, during which activity was recorded. 30 μL of 550 μM GABA was added to the 300 μL of cell media in each culture for a final GABA concentration of 50 μM. Uninhibited motor neuron networks were only supplemented with 30 μL of cell media with no GABA. The experiment therefore had four groups in total: inhibited hypoxic (*n* = 12), uninhibited hypoxic (*n* = 12), inhibited normoxic (*n* = 11), and uninhibited normoxic (*n* = 10). Prior to the experiment, baseline activity was recorded for all neural networks for 1 h. After the hypoxia/control exposure, half media changes were made to all neural networks. Starting the day after the hypoxic challenge, activity was recorded for 1 h daily for 10 days, and half of the media volume was replenished every other day. The last recordings were made at 64 DIV. A more detailed timeline of the hypoxic challenge is shown in Figure 1B.

### Data Analysis

Network bursts were identified by examining a binned spike distribution using 50 ms bins. Every bin that exceeded a threshold of firing rate was identified as potentially part of a burst. The threshold used was mean plus 5 standard deviations, indicated in red in Figure 1C. Then, the number of firing electrodes in each of these bins was checked, and bins with high firing rate but fewer than 20% of active electrodes in the recording fired were considered non-bursting activity. Finally, subsequent time bins with bursting were combined into longer bursts. From this burst train, identifying burst beginnings and ends as well as size in terms of participating electrodes, further measures were derived, such as the proportion of spikes which occurred in network bursts, hereafter referred to as burst propensity. Network synchrony was measured by the coherence index.

Because the data was not normally distributed, all summary data is shown as median ± median absolute deviation and comparisons between groups were assessed by Wilcoxon rank sum test, with Bonferroni correction for multiple comparisons. Relative dispersion of the data was assessed by an alternative to the coefficient of variation based on interquartile range, RCV_*Q*_ (Arachchige et al., 2020).

## Results

### Immunocytochemistry

**FIGURE 2:**
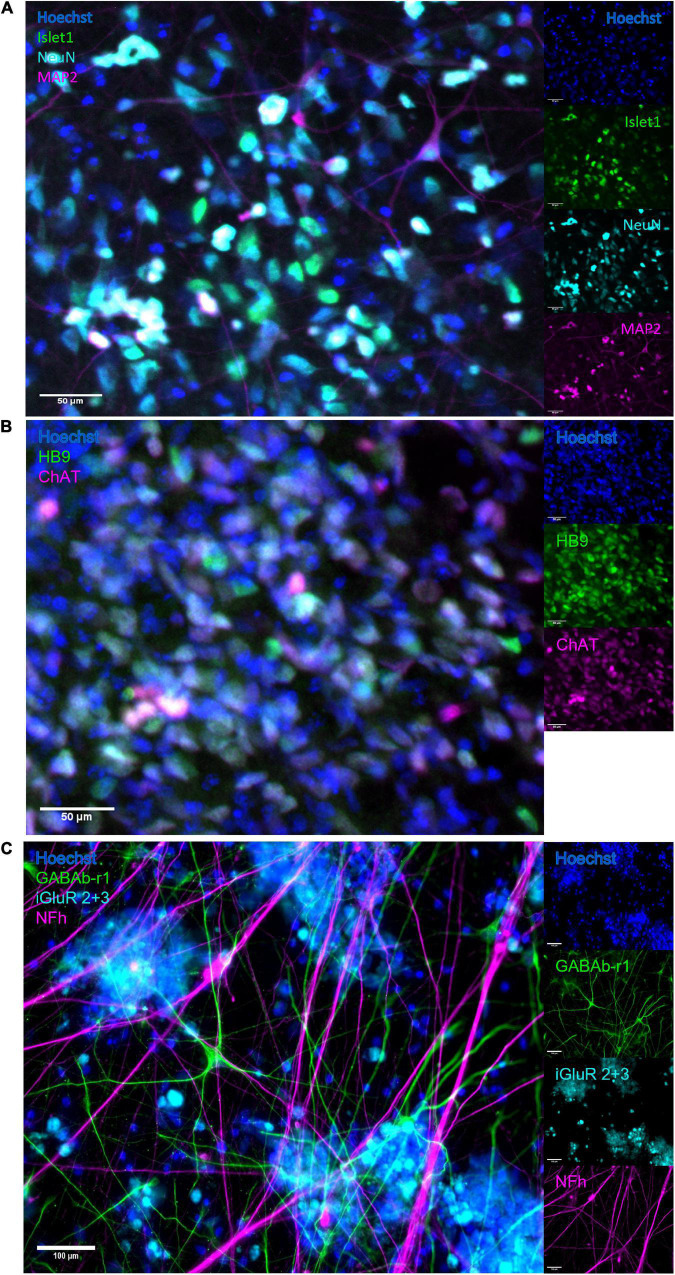
Immunocytochemistry. After maturation, neurons expressed motor neuron specific markers as well as relevant neurotransmitter receptors and markers of mature cytoskeleton. **(A)** Overlap of expression of motor neuron specific marker Islet1 and neuronal marker NeuN, alongside heavy neurofilament, indicates the presence of mature motor neurons. Scale bar 50 μm. **(B)** The co-expression of motor neuron markers HB9 and ChAT further confirms motor neuron identity. Scale bar 50 μm. **(C)** Expression of receptors for GABA and glutamate confirms the capacity for excitatory signalling within the motor networks, as well as the susceptibility of the networks to the GABA-inhibition. Scale bar 100 μm.

The neural networks were imaged live throughout development until 49 DIV, and matured from a dissociated, even layer of cells into progressively more aggregated clusters connected by fasciculated neurites. After maturation at 49 DIV, immunostaining confirmed expression of GABA and glutamate receptors as well as heavy neurofilament, a mature cytoskeletal marker (Figure 2C) and motor neuron markers Islet 1 (Figure 2A), HB9 and ChAT (Figure 2B).

### Neural Network Activity and Response to Hypoxic Challenge

**FIGURE 3:**
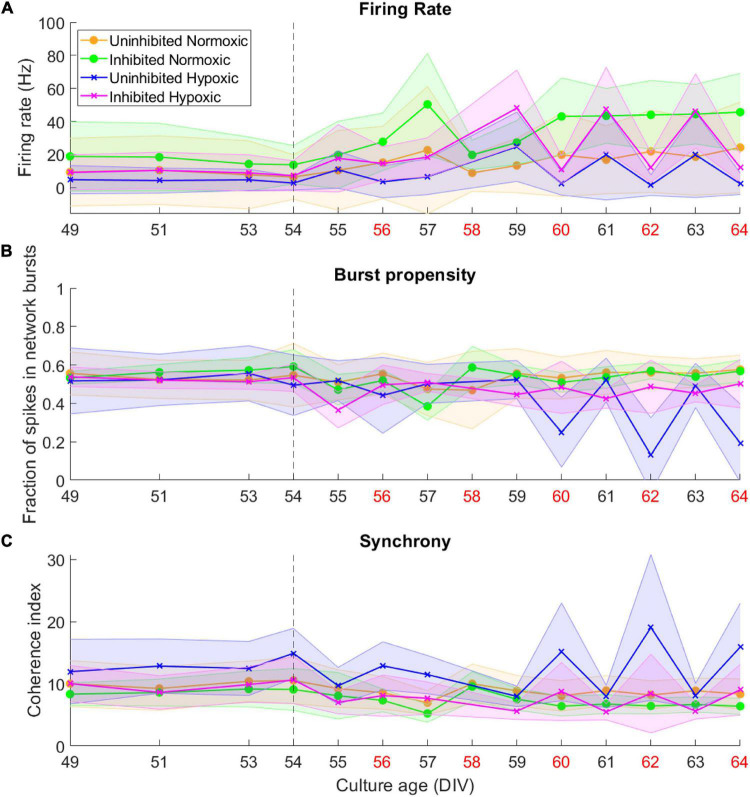
Activity of neural networks before and after the hypoxic challenge. The activity of the motor neuron networks is described in terms of firing rate **(A)**, fraction of spikes in network bursts, or bursting propensity **(B)**, and coherence index, a measure of network synchrony **(C)**. The stippled line at 54 DIV indicates when the hypoxic challenge was carried out, and the data points presented for this day are baseline recordings pre-hypoxia. Red labels along the Culture Age axis indicates that cell media was replaced these days. Lines and shaded regions indicate median ± median absolute deviation.

By 49 DIV, the motor neuron networks had largely reached a stable state in terms of activity, burst propensity and synchrony. Following the hypoxic challenge at 54 DIV, activity remained fairly stable for all groups until 59–60 DIV. The normoxic networks remained stable after this point and throughout the recording period. The hypoxic networks, however, exhibited fluctuations in activity after this point. Both inhibited and uninhibited hypoxic networks showed consistent changes in firing rate from day to day (Figure 3A), alternating between high (inhibited 47.27 ± 0.90 Hz, uninhibited 21.42 ± 2.80 Hz) and low (inhibited 11.61 ± 0.76 Hz, uninhibited 2.00 ± 0.54 Hz). Burst propensity and synchrony were different between the inhibited and the uninhibited hypoxic networks. The inhibited networks maintained entirely stable burst propensity comparable to control networks, while the uninhibited networks fluctuated in this regard in a similar way as seen in firing rate (Figure 3B). The uninhibited and inhibited hypoxic networks both showed similar fluctuations from low to high synchrony in co-occurrence with the fluctuations in firing rate and burst propensity, although in an opposite manner (Figure 3C).

**FIGURE 4:**
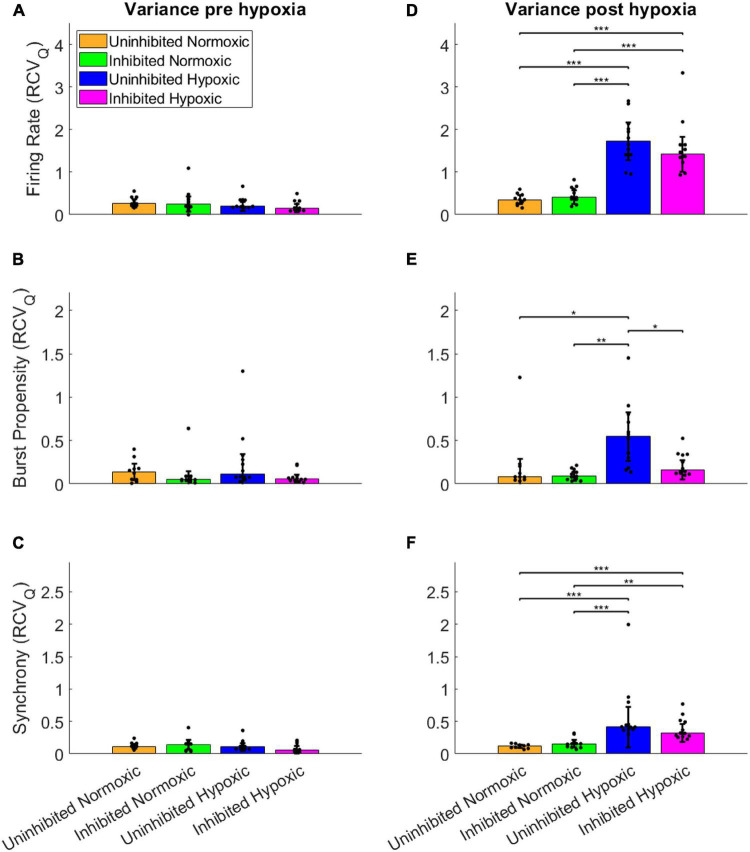
Dispersion in network activity pre and post the hypoxic challenge. The dispersion of the electrophysiology parameters was assessed by the RCV_Q_ of the activity of each network and compared by Wilcoxon rank sum test, prior to and following the hypoxic challenge. The longitudinal dispersion pre-hypoxia showed no significant differences in terms of firing rate **(A)**, bursting propensity **(B)**, or synchrony **(C)**. Post-hypoxia the hypoxic networks showed similar significant increases in longitudinal dispersion in terms of firing rate **(D)** and synchrony **(F)**. However, only the uninhibited hypoxic networks showed a significant increase in the dispersion of bursting propensity, while the inhibited hypoxic networks showed significantly less longitudinal dispersion, in line with control networks **(E)**. *p < 0.0083, **p < 0.0017, ***p < 0.00017. Bars and error bars indicate median ± median absolute deviation.

To assess the fluctuations of the network activity, the relative dispersion in the longitudinal data for each network was evaluated by RCV_*Q*_ prior to and after the hypoxic challenge. The results of the statistical tests are shown in Table 2. Pre-hypoxia, there was no significant difference between the longitudinal dispersion of any electrophysiology parameter across the experimental groups, as seen in Figures 4A–C. As shown in Figure 4D, the hypoxic networks subsequently exhibited significant increases in dispersion of firing rate compared to normoxic networks, regardless of inhibition status (*p* < 0.0083). A similar relationship was observed in dispersion of synchrony following hypoxia, as seen in Figure 4F, which was also seen regardless of inhibition status (*p* < 0.0083). The dispersion in burst propensity following hypoxia was significantly increased in uninhibited hypoxic networks compared to normoxic control networks (*p* < 0.0083), seen in Figure 4E. However, there was no significant difference between the dispersion in burst propensity of normoxic control networks and inhibited hypoxic networks. Furthermore, the latter group showed significantly less dispersion in burst propensity compared to uninhibited hypoxic networks, suggesting that GABA inhibition contributed to maintained bursting activity in motor neuron networks.

**TABLE 2:** Statistical comparison of network activity.

Firing rate	Uninhibited	Inhibited	Uninhibited
pre-hypoxia	normoxic	normoxic	hypoxic
**Uninhibited normoxic**			
Inhibited normoxic	0,53		
Uninhibited hypoxic	0,31	0,89	
Inhibited hypoxic	0,019	0,14	0,030

**Burst propensity**	**Uninhibited**	**Inhibited**	**Uninhibited**
**pre-hypoxia**	**normoxic**	**normoxic**	**hypoxic**

**Uninhibited normoxic**			
Inhibited normoxic	0,22		
Uninhibited hypoxic	0,53	0,053	
Inhibited hypoxic	0,34	0,52	0,026

**Synchrony**	**Uninhibited**	**Inhibited**	**Uninhibited**
**pre-hypoxia**	**normoxic**	**normoxic**	**hypoxic**

**Uninhibited normoxic**			
Inhibited normoxic	0,81		
Uninhibited hypoxic	0,87	0,78	
Inhibited hypoxic	0,12	0,44	0,053

**Firing rate**	**Uninhibited**	**Inhibited**	**Uninhibited**
**post-hypoxia**	**normoxic**	**normoxic**	**hypoxic**

**Uninhibited normoxic**			
Inhibited normoxic	0,13		
Uninhibited hypoxic	8,73⋅10^–05^	5,55⋅10^–05^	
Inhibited hypoxic	8,73⋅10^–05^	5,55⋅10^–05^	0,19

**Burst propensity**	**Uninhibited**	**Inhibited**	**Uninhibited**
**post-hypoxia**	**normoxic**	**normoxic**	**hypoxic**

**Uninhibited normoxic**			
Inhibited normoxic	0,81		
Uninhibited hypoxic	0,0051	0,00040	
Inhibited hypoxic	0,044	0,013	0,0073

**Synchrony**	**Uninhibited**	**Inhibited**	**Uninhibited**
**post-hypoxia**	**normoxic**	**normoxic**	**hypoxic**

**Uninhibited normoxic**			
Inhibited normoxic	0,22		
Uninhibited hypoxic	8,73⋅10^–05^	9.30⋅10^–05^	
Inhibited hypoxic	8,73⋅10^–05^	0,00080	0,069

*Statistical comparisons were performed on the relative dispersion of network parameters for each network before and after the hypoxic challenge or normoxic control, comparing the different groups by Wilcoxon rank sum test. With Bonferroni correction, differences were considered significant at p < 0.0083.*

In summary, the uninhibited hypoxic networks demonstrated changes in activity which only became evident 5–6 days after the hypoxic challenge, consisting of alternating decreases in firing rate and burst propensity and increases in network synchrony. The inhibited hypoxic networks on the other hand, showed the same fluctuations in firing rate and synchrony, but not in burst propensity. Normoxic control networks, did not exhibit any fluctuations regardless of inhibition status. The fluctuations led to significant increases in longitudinal dispersion of the network activity parameters for the hypoxic networks, with the exception of the dispersion in burst propensity of the inhibited hypoxic networks, which remained similar to the levels seen in normoxic control networks and significantly lower than uninhibited hypoxic networks.

Of note is the timing of the fluctuations in terms of media changes in handling the networks. Points of high firing rate occurred closer to the previous media change (about 24 h), while points of low firing rate coincided with longer time elapsed since the previous media change, indicated by red *x*-axis labels in Figure 3 (about 48 h).

### Hypoxic Challenge

**FIGURE 5:**
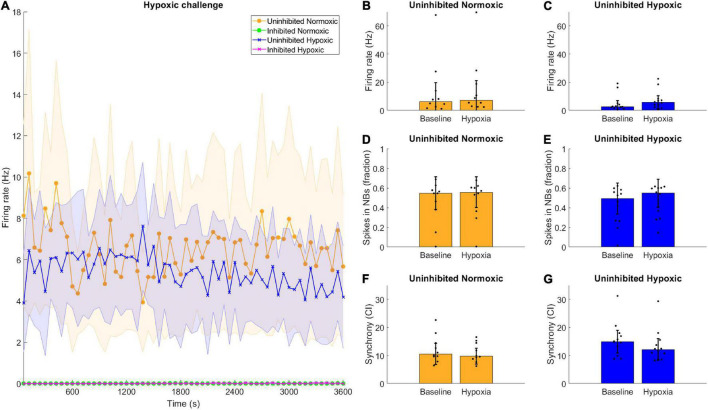
Hypoxic challenge and GABA inhibition. The hypoxia exposed, uninhibited networks showed similar activity to uninhibited networks normoxic networks, while inhibited networks were completely silenced. **(A)** Data during the hypoxia session was divided into 1-min bins and is shown here throughout the 1-h recording. Uninhibited networks showed stable activity throughout the episode, while inhibited networks were completely silenced. Lines and shaded regions indicate median ± median absolute deviation. **(B–G)** There were no significant differences between the baseline electrophysiological activity and the activity during the hypoxic exposure in terms of firing rate, burst propensity or synchrony, for normoxic controls or hypoxic networks, assessed by Wilcoxon rank sum test, all *p* > 0.05. Bars and error bars indicate median ± median absolute deviation.

Neuronal activity was recorded during hypoxia, and there were no clear differences observed between the hypoxic and the normoxic networks in terms of firing rate. All the inhibited networks remained quiet, with firing rate near or at zero throughout the recording period (Figure 5A). Furthermore, there were no clear differences between the activity displayed during the baseline observations and the hypoxic challenge, for either normoxic controls or hypoxic networks, in any assessed network activity parameter (Figures 5B–G).

## Discussion

By the time the motor neuron networks in this study were mature at 49 DIV they exhibited stable activity in terms of firing rate, a high degree of burst propensity and stable levels of synchrony as seen up to 54 DIV in Figure 3, which are in line with observations for functionally mature networks (Pasquale et al., 2008). These networks also expressed markers of mature neuron cytoskeletons and relevant receptors including GABAb-1 receptors, shown in Figure 2C, as well as specific markers for motor neurons, shown in Figures 2A,B.

Interestingly, the hypoxic networks showed no changes in activity during the episode itself compared to the normoxic controls. There were no discernible differences in firing rate, burst firing or synchrony in the uninhibited hypoxic networks compared to the pre-hypoxia baseline (Figures 5C,E,G). The same was predictably true for uninhibited normoxic networks (Figures 5B,D,F). Previous reports have indicated that exposure to hypoxia can lead to acute hyperexcitability leading to elevated firing rate in hypothalamic neurons (Shonis and Waldrop, 1995) while others have found that chemical hypoxia leads to reduced excitability (Englund et al., 2004), although investigations of ongoing activity during hypoxia rather than post-hypoxic recovery are rare, especially in motor neurons. Such changes in activity were not observable in the current motor neuron networks, and changes in activity following exposure to hypoxic challenge only became evident later on.

It should be noted that the astrocytes co-cultured in the motor neuron networks may provide neuroprotective effects to the motor neurons in this study, a widely reported phenomenon (Marina et al., 2016). However, since all experimental groups contained the same ratio of neurons to astrocytes, and evidence suggests that astrocytes are more resilient than neurons to hypoxic conditions (Alves et al., 2000), it seems unlikely that potential effects of astrocytes is a major contributing factor to the observed group differences.

This study demonstrated that mature networks of motor neurons exposed to hypoxic challenge exhibit aberrant fluctuations in network activity which were not evident in networks maintained in normoxic conditions, seen clearly in Figure 3. The fluctuations occurred over the course of 59–64 DIV, and involved changes from high to low firing rate, from normoxic levels of burst propensity to low levels, and from normoxic levels of network synchrony to elevated levels. This loss of homoeostasis and stable network function differs significantly from the normoxic networks, as seen in Figure 4, showing the detrimental effects of the hypoxic exposure on network function. Interestingly, this effect did not become apparent until 5–6 days following the hypoxic challenge, as seen in Figure 3. This may suggest that there are compensatory mechanisms or adaptive responses which can maintain function close to that of normoxic counterparts, but that these measures are only effective for a limited time.

Furthermore, the timing of the observed changes in activity follow a very characteristic pattern, with deviations from normoxic-like levels observed on alternate days which correspond to observations immediately prior to cell media replacement, meaning that more of the nutrients in the cell media were depleted compared to the observations where network activity was more similar to that of normoxic networks. It is possible that this is a result of an increased metabolic burden due to effects of the hypoxia and any homeostatic mechanisms which fail to maintain as nutrients are depleted. High metabolic requirements are seen across multiple populations of neurons vulnerable to neurodegenerative disease (Muddapu et al., 2020) and may contribute to their selective vulnerability. Similarly, during hypoxia, due to the uncoupling of oxidative phosphorylation, the production of ATP becomes less efficient, eventually leading to elevated oxidative stress (Jain et al., 2015; Snyder et al., 2017; Chen et al., 2018). The effects of hypoxia can thus be more adverse for neuronal populations with high energy requirements. The results from this study may therefore suggest that motor neuron networks exposed to hypoxia exhibit increased metabolic stress, which leads to loss of functional homoeostasis and could be a predisposing factor for further network degeneration.

Some of the changes in network activity appear to be related to activity during the hypoxic challenge, since inhibition of the network with GABA improves functional outcomes in terms of maintaining network burst propensity. While inhibition of the normoxic networks appeared to have no lasting effect, the hypoxic networks inhibited during the hypoxic challenge exhibited normoxic levels of burst propensity throughout the experiment (Figures 3B, 4E), despite exhibiting similar fluctuations as the uninhibited hypoxic networks in terms of the other activity parameters (Figures 3A,C, 4D,F). Network bursts are an emergent property of activity of networks of neurons and is considered to be more reliable in terms of signalling and can carry more information (Zeldenrust et al., 2018). The maintenance of this parameter in the silenced hypoxic networks indicates improved functional outcomes as a result of the silencing, even though there are still clear consequences of the hypoxic challenge. It is possible that there are activity-dependent mechanisms which occur during hypoxia which lead to a delayed loss of function, either due to plasticity-induced changes during the episode or due to metabolic stress incurred by maintaining activity during hypoxic challenge. Acute hypoxia like in this study has been reported to activate HIF-1 through mitochondria-dependent mechanisms, a function of which is to upregulate anaerobic respiration and inhibiting the oxygen dependent TCA cycle, which places the affected cells in an energy-deficient state (Solaini et al., 2010; Hernandez-Gerez et al., 2019). From the results of our study, it appears that the activity-dependent damage was due to maintenance of normal activity, indicated by the results in Figure 4, rather than due to changes in activity during the hypoxic challenge. Maintaining activity during such circumstances of less energy production can lead to increased production of ROS which have the potential to cause damage to the network. Elevated levels of oxidative stress markers have previously been reported in mouse models of ALS exposed to hypoxic conditions (Kim et al., 2013). It seems likely that inhibition the networks during the hypoxic challenge would reduce the energy requirements of the motor neurons, thus reducing the metabolic stress. Inhibiting the networks contributed to maintaining pre-hypoxic functionality in terms of network burst propensity, although the persistent fluctuations in firing rate and synchrony indicate that the hypoxic challenge had effects on the networks which were not dependent on activity during the hypoxic challenge itself.

Although there are conflicting reports on the immediate effects of hypoxia exposure on network activity, it is evident that the mechanisms involved, at least in part, increased oxidative damage through calcium dysregulation (Kristian and Siesjo, 1998; Starkov et al., 2004). In this context, there is a particular association with the activity of NMDA-receptors and findings suggest that blocking them can reduce the production of ROS during hypoxia (Lafon-Cazal et al., 1993) as well as ameliorate post-hypoxic hyperexcitability (Godukhin et al., 2002). When addressing hypoxia and considering interventions it is vital to know what pathways can be targetted in order to improve outcomes for patients, for example those who require assisted ventilation, which can be associated with acute hypoxic events (Keith and Pierson, 1996; Mahmood et al., 2013). This study suggests that functional outcomes can be preserved by targetting activity-dependent pathways.

The study of hypoxia and the mechanisms by which it damages neural networks bears a direct relevance to neurodegenerative diseases like ALS. The body of evidence, which shows that mutations in hypoxia-responsive elements like angiogenin (ANG) can increase the likelihood of developing ALS (Greenway et al., 2006; van Es et al., 2011; Zou et al., 2012), indicates that some mechanisms of healthy responses to hypoxia are also involved in the ongoing health of motor neurons which fail in ALS. Additionally, SOD1 genetic models of ALS exhibit altered responses to hypoxia, which may exacerbate their susceptibility to hypoxic stress (Cimini et al., 2014), and elevated levels of hypoxia responsive elements including ANG, apoptosis inducing factor and HIF-1α (Zhang et al., 2011) are observed in both ALS patients (Cronin et al., 2006; van Es et al., 2014; Morelli et al., 2020) and mouse models (Xu et al., 2011). Furthermore, exposure to repeated hypoxic episodes appears to increase the risk of developing ALS (Vanacore et al., 2010), and studies in mouse models of ALS have found that stabilising HIF-1α, in effect enhancing the hypoxic response mechanism, reduced degeneration of spinal motor neurons and myofibrils of the lower limbs, as well as increasing the lifespan of the mice (Nomura et al., 2019). Mouse models of ALS exposed to hypoxic conditions also exhibit accelerated disease progression compared to normoxic controls (Kim et al., 2013). These findings suggest a relationship between the mechanisms of resilience to hypoxia and the risk of developing ALS as well as the severity of the disease progression. The results of this study indicate that there are activity-dependent mechanisms which lead to network dysfunction following hypoxia. Not unexpectedly, studies of ALS show that activity-dependent processes, hyperexcitability and formation of ROS is directly linked with motor neuron damage (Kaus and Sareen, 2015; Verma et al., 2018; Marcuzzo et al., 2019). ALS has also been observed to alter cortical hub regions, i.e., highly interconnected structures which are vital for proper network function, early on in the disease process (Verstraete et al., 2011; Ma et al., 2015). Given that cortical hubs are known to be highly metabolically demanding (Bullmore and Sporns, 2012; Collin et al., 2014), it is natural to consider that they might be some of the first parts of the neural network to show deterioration in circumstances where there is reduced capacity to respond healthily to activity-dependent adversity, such as a reduction in functionality of the hypoxia-responsive machinery.

In conclusion, this study examined the responses of motor neuron networks to a hypoxic challenge and how the functionality of the networks develops over time, with and without inhibition of activity during the challenge. We found that although activity during the hypoxic challenge remained similar to both baseline measurements and to normoxic controls, hypoxic networks began to show clear deviations from the behaviour of normoxic networks, involving fluctuations in activity. Inhibition of network activity during the hypoxic challenge contributed to preserved bursting behaviour compared to uninhibited networks, an improved functional outcome. These responses could not have been captured without a longitudinal assessment of the network activity, which is rarely seen in the existing literature. Elucidating patterns of dysfunction following hypoxic challenge and the mechanisms involved has implications beyond the hypoxia itself, such as in diseases like ALS, where the mechanisms of degeneration appear to be at least in part shared with those observed in hypoxia. Assessing these mechanisms can thus contribute to a more precise investigations of these properties in a highly multifactorial neurodegenerative disorder.

## Data Availability Statement

The raw data supporting the conclusions of this article will be made available by the authors, without undue reservation.

## Author Contributions

VF conceived of and designed the study, acquired the data, performed the statistical analysis, and drafted the manuscript. AS and IS participated in the design of the study and helped to revise the manuscript. All authors read and approved the final version of the manuscript.

## Conflict of Interest

The authors declare that the research was conducted in the absence of any commercial or financial relationships that could be construed as a potential conflict of interest.

## Publisher’s Note

All claims expressed in this article are solely those of the authors and do not necessarily represent those of their affiliated organizations, or those of the publisher, the editors and the reviewers. Any product that may be evaluated in this article, or claim that may be made by its manufacturer, is not guaranteed or endorsed by the publisher.
